# Clinical benefits of *Bifidobacterium infantis* YLGB-1496 in modulating gut microbiota and immunity in young children

**DOI:** 10.3389/fnut.2025.1713135

**Published:** 2026-01-05

**Authors:** Mageswaran Uma Mageswary, Pin Li, Rocky Vester Richmond, Yusof Azianey, Intan Juliana Abd Hamid, Fahisham Taib, Min-Tze Liong, Adli Ali, Joo Shun Tan, Yumei Zhang

**Affiliations:** 1 School of Industrial Technology, Universiti Sains Malaysia, Gelugor, Pulau Pinang, Malaysia; 2 Department of Nutrition and Food Hygiene, School of Public Health, Peking University Health Science Center, Beijing, China; 3 Department of Pediatrics, Universiti Kebangsaan Malaysia Medical Centre, Faculty of Medicine, Universiti Kebangsaan Malaysia, Kuala Lumpur, Malaysia; 4 Kepala Batas Health Clinic, Kepala Batas, Pulau Pinang, Malaysia; 5 Department of Clinical Medicine, Advanced Medical & Dental Institute, Universiti Sains Malaysia, Kepala Batas, Pulau Penang, Malaysia; 6 Paediatric Department, School of Medical Sciences, Universiti Sains Malaysia, Kubang Kerian, Kelantan, Malaysia; 7 Research Center, Hospital Tunku Ampuan Besar Tuanku Aishah Rohani, Universiti Kebangsaan Malaysia Specialist Children's Hospital, Kuala Lumpur, Malaysia

**Keywords:** probiotic, children, respiratory tract infections, gut microbiota, randomized controlled trial, *Bifidobacterium infantis* YLGB-1496

## Abstract

**Introduction:**

The early-life gut microbiota is critical for immune development and long-term health, and plays an essential role in the digestion and metabolism of dietary components, including human milk oligosaccharides (HMOs). Probiotic supplementation is a promising strategy to modulate this ecosystem and prevent common childhood infectious illnesses, though strain-specific effects require further investigation.

**Method:**

This 12-week, double-blind, randomized, placebo-controlled trial evaluated the efficacy of *Bifidobacterium infantis* YLGB-1496 (1 × 1010 CFU/day) in 119 healthy preschool children. Participants were assessed for respiratory and gastrointestinal (GI) illness incidence, inflammatory biomarkers (fecal IgA, cytokines, calprotectin; salivary cortisol), and gut microbiota composition via 16S rRNA sequencing.

**Result:**

Probiotic supplementation significantly reduced the incidence of respiratory problems as compared to the placebo group (Week 12: 15.0 vs. 42.4%, *p* < 0.001) and diarrhea (Week 6: 18.3 vs. 44.1%, *p* = 0.002), alongside fewer clinical visits and antibiotic prescriptions (*p* < 0.01 for all). Immunologically, the probiotic group exhibited a favorable anti-inflammatory profile with reduced levels of fecal IFN-γ, IL-1β, and calprotectin, and a trend toward increased fecal IgA over time as compared to the placebo group. Microbiota analysis revealed that the probiotic did not induce major restructuring but provided ecological stability, preventing the and preserving beneficial SCFA producing genera that declined in the placebo group (*p* < 0.05).

**Conclusion:**

*B. infantis* YLGB-1496 is an effective probiotic that reduces the burden of common childhood infectious illnesses by fine-tuning immune responses and enhancing the resilience of the gut microbial ecosystem, rather than through drastic compositional changes. These findings support its use as a safe nutritional intervention for promoting pediatric health.

**Clinical trial registration:**

https://clinicaltrials.gov/study/NCT05794815?term=NCT05794815&rank=1, identifier number: NCT05794815.

## 1 Introduction

The gastrointestinal tract harbors a highly diverse microbial ecosystem, collectively termed the gut microbiota, which is integral to host metabolism, immune maturation, nutrient digestion, and protection against pathogens (1). In children, the early establishment and progressive maturation of the gut microbiota are particularly critical, as this developmental window shapes immune homeostasis, nutrient absorption, including the breakdown of complex dietary polysaccharides such as human milk oligosaccharides (HMOs) and long-term health outcomes (2). Bifidobacteria, especially in infancy, are known to play a pivotal role in the utilization of HMOs, contributing to energy harvest and the production of short-chain fatty acids (SCFAs) that support gut health (3). Perturbations in the pediatric gut microbiota arising from factors such as antibiotic exposure, dietary transitions, or environmental influences that have been associated with increased risks of gastrointestinal infections, allergic diseases, obesity, and metabolic disorders (4, 5).

Probiotics, defined as “live microorganisms which, when administered in adequate amounts, confer a health benefit on the host” (6), have emerged as a promising approach to beneficially modulate the gut microbiota. Several clinical and experimental studies have demonstrated that specific probiotic strains enhance microbial diversity, restore microbial equilibrium, and promote colonization by beneficial taxa such as lactobacilli and bifidobacteria (7). Moreover, probiotics exert functional effects beyond compositional shifts, including reinforcement of gut barrier integrity, production of short-chain fatty acids (SCFAs), and modulation of mucosal and systemic immune responses (8). In pediatric populations, probiotics have been reported to reduce the duration of acute infectious diarrhea, lower the incidence of antibiotic-associated diarrhea, attenuate allergic manifestations, and support overall gut health (9, 10).

Among the various probiotic genera, Bifidobacterium is one of the most predominant and earliest colonizers of the human infant gut, playing a fundamental role in the development of the gut ecosystem and immune system (2). Consequently, bifidobacteria have been extensively studied and applied as probiotics in pediatric populations. Specific strains of Bifidobacterium have demonstrated efficacy in reducing the risk and duration of infectious diarrhea and antibiotic-associated diarrhea in children (11). Furthermore, certain bifidobacterial strains have shown promise in alleviating symptoms of infant colic and atopic eczema, and in providing protection against respiratory tract infections (12, 13). The safety profile and these documented health benefits make Bifidobacterium a key genus for developing probiotic interventions aimed at supporting child health.

Despite these benefits, probiotic effects remain strain-specific, dose-dependent, and influenced by host factors such as age, diet, and baseline microbiota composition (14). Furthermore, while clinical outcomes have been relatively well-studied, comprehensive insights into how probiotics modulate the gut microbial structure and function in children remain limited. Addressing this knowledge gap is essential to advance targeted probiotic applications and to inform evidence-based recommendations for pediatric health.

The present study investigates the impact of probiotic supplementation on the gut microbiota of children over a 12-week intervention. By integrating microbial diversity analyses and taxonomic profiling, this work aims to elucidate the extent and specificity of microbiota modulation induced by probiotics, thereby contributing to a mechanistic understanding of their role in pediatric gut health.

## 2 Materials and methods

### 2.1 Probiotic and placebo products

Probiotic (*Bifidobacterium infantis* YLGB-1496) which isolated from breast milk (15) and placebo products appeared as light-yellow powder and were kept at storage temperature range below 25 °C according to the condition recommended by the manufacturer. The probiotic product contained probiotic and carrier while placebo contained only carrier. The intervention consisted of orally administered one sachet/day of probiotic (1 × 10 log CFU/sachet) or placebo, for 12-weeks.

### 2.2 Study population

This was a double-blind, randomized, placebo-controlled design study. Randomization was conducted upon considering the inclusion and exclusion criteria. Qualified subjects were randomized according to 1:1 ratio to the two arms of the study according to a computer-generated list, assigned to the probiotic or placebo group with individual codes. Randomization was performed by the study statistician, who had no contact with the participants. The allocation sequence was not available to any member of the research team until the completion of the study. This study was conducted according to the Declaration of Helsinki where all procedures involving human subjects were approved by the UKM Research Ethics Committee (approval number UKM/PPI/111/8/JEP-2023-972; approval date 29 May 2024) and was registered at ClinicalTrials.gov (identifier number NCT05794815) on 20th March 2023.

The sample size was calculated for a parallel group study design involving one prevention arm and one placebo arm and was based on power design analysis. A total of 120 subjects is needed for this study, comprising of 60 total subjects in each group, including an additional 10% dropout (*n* = 54 in each group without dropout). This calculation was based on the need for a continuous response variable from independent control and experimental subjects, with a ratio of control to subject fixed at 1:1, probability of 0.95 and Type-I error probability associated with this test of null hypothesis of 0.05. Previous data have shown that for an intervention using probiotic against reducing clinical visits in children for respiratory diseases, a standard deviation of 0.46 times within group was observed, accompanied by a reduction of 0.32 times between treatment and placebo groups (16).

### 2.3 Study protocol

Written informed consent was obtained from all participants prior to the start of the study. Subjects were recruited from the Universiti Kebangsaan Malaysia (UKM) campus as well as community engagements. Inclusion criteria included preschool children, healthy with current weight of P20–P80 percentile chart of children, currently consuming infant formula powder that does not contain probiotics, with prebiotics such as Fructooligosaccharide (FOS) is not allowed while Galactooligosaccharide (GOS) is allowed at concentration < 2/100 g, and willing to commit throughout the experiment. Exclusion criteria included those on long term medication (>6 months) for any diseases, having any deformity, with mothers having metabolic and/or chronic diseases, current or previous diseases, conditions or interventions that may interfere with the study (such as tolerance and/or growth and development), and such as gastrointestinal malformations, chronic diarrhea, malabsorption syndromes, malnutrition, congenital immunodeficiency or surgery, having consumed oral antibiotics within 2 weeks before the intervention, consumed nutritional supplements containing probiotics and prebiotics (except infant formula) within 2 weeks before the intervention, consuming foods for special medical purposes or non-standard formula powders for lactose intolerance and galactosemia, having known probiotic allergies or possible food allergies, participated in other clinical studies 4 weeks before the intervention, or unwilling to participate for any particular reason.

### 2.4 Questionnaires

Eligible subjects were given a demographic questionnaire at baseline (week 0). At baseline, week 6, and week 12, parents or guardians completed a detailed symptom and healthcare utilization questionnaire. This instrument was a simplified, Malay-language translated version of a validated questionnaire used in previous pediatric probiotic studies (17). Respiratory health and gastrointestinal health were monitored based on parent-reported symptoms and healthcare utilization using the validated questionnaire. Parents were asked to record the presence and duration of predefined respiratory symptoms such as cough, runny nose, fever, and sore throat (18). A diarrheal episode was defined by Bristol stool chart. The questionnaires captured the incidence, frequency, and duration of diarrhea, stool characteristics, the presence and duration of associated GI symptoms such as vomiting, abdominal pain, fever, and related healthcare utilization (clinical visits and medications prescribed).

### 2.5 Sample collection and immunological analysis

At weeks 0, 6, and 12, oral swab samples were collected from each toddler from the inside of both the left and right cheeks, one immediately after the other. At the same time points, fecal samples were also collected in fecal collection tubes and glass beads by the subjects. All the tubes were capped tightly and stored at −80 °C until further analyses. These samples were collected by our medical team.

The fecal samples were analyzed for the concentrations of cytokines, including Ig A, calprotectin, α-1-antitrypsin, tumor necrosis factor-α (TNF-α), interferon-γ (IFN-γ), interleukin-1β (IL-1β), IL-4, IL-6, and IL-10. The oral swab samples were collected for the analysis of cortisol and Ig A. Fecal samples were homogenized in phosphate-buffered saline, centrifuged (3,000 × rpm, 20 min, 4 °C), and the resulting supernatants were collected for analysis. Oral swab samples were eluted in PBS by vertexing for 30 min, followed by centrifugation to collect the eluent.

The quantification of fecal cytokines was selected to provide a direct measurement of the inflammatory milieu at the intestinal mucosal surface, the primary interface for host-microbiome and host-probiotic interaction. While systemic cytokine levels reflect a global immune status, fecal cytokines offer a more localized and physiologically relevant assessment of gut-specific immune activity (19, 20). We acknowledge the technical challenges associated with their measurement in stool, including potential degradation. To mitigate this and ensure analytical reliability, a rigorous protocol was implemented: all fecal samples were immediately stabilized at −80 °C after collection to halt protease activity, and subsequent processing (homogenization, centrifugation) was standardized across all samples. Furthermore, all analyses were conducted using commercial ELISA kits specifically validated for the complex fecal matrix. The biological validity of this approach is supported by the coherent patterns observed in our data, where changes in fecal cytokines aligned with those of the stable inflammatory marker calprotectin and the reported clinical outcomes.

The concentrations of immunological biomarkers in the supernatants and eluents were quantified using specific commercial sandwich Enzyme-Linked Immunosorbent Assay (ELISA) kits (Sunlong Biotech Co., Ltd., Hangzhou, Zhejiang, China), strictly following the manufacturer's protocols. The absorbance was measured using a microplate reader (Multiskan Go, Thermo Fisher Scentific, Waltham, MA, USA).

### 2.6 Microbiota analyses

For microbiota analysis, aliquots of the same fecal samples collected at week 0, 6, and 12 (as described in Section 2.5) were immediately placed into sterile fecal collection tubes containing RNAlater™ Stabilization Solution (Thermo Fisher Scientific, Waltham, MA, USA) to preserve microbial DNA and stored at −80 °C until DNA extraction.

DNA extraction and purification from fecal samples were performed as previously described (21). Purified DNAs were determined by NanoDrop 2000 UV-Vis Spectrophotometer (Thermo Fisher Scientific, Waltham, MA, USA). The V3–V4 hypervariable regions of the bacteria 16S rRNA gene were amplified with primers 341F (5′-CCTAYGGGRBGCASCAG-3′) and 806R (5′-GGACTACHVGGGTWTCTAAT-3′) by thermocycler PCR system (GeneAmp 9700, Applied Biosystems, Foster City, CA, USA). The PCR reactions were performed in triplicates with 20 μl mixture containing 4 μl of 5 × FastPfu Buffer, 2 μl of 2.5 mM dNTPs, 0.8 μl of each primer (5 μM), 0.4 μl of FastPfu Polymerase, and 10 ng of template DNA, in the following sequence: 3 min of denaturation at 95 °C, 27 cycles of 30 s at 95 °C, 30 s for annealing at 55 °C, and 45 s for elongation at 72 °C, and a final extension at 72 °C for 10 min. The PCR products were then extracted from a 2% agarose gel and further purified using the AxyPrep DNA Gel Extraction Kit (Axygen Biosciences, Corning, NY, USA) and quantified using QuantiFluor™-ST (Promega Corporation, Madison, WI, USA) according to the manufacturer's protocol. The purified amplicons were pooled in equimolar and paired end sequenced (2 × 300) on an Illumina MiSeq platform (Illumina, Inc., San Diego, CA, USA).

The 16s rRNA gene sequences were processed using QIIME v.1.9.1 (ref QIIME allows analysis of high-throughput community sequencing data) and USEARCH v.10.0 (ref Search and clustering orders of magnitude faster than BLAST). Raw FASTQ files were quality filtered by Trimmomatic and merged by USEARCH with the following criteria: removing of barcodes and primers, filtering of low-quality reads, and finding non redundancy reads. The merged raw reads were at least 50,000 per sample. Operational taxonomic units (OTUs) were clustered with 97% similarity cut-off using UPARSE. The taxonomy for each 16S rRNA gene sequence was analyzed by the RDP Classifier algorithm (http://rdp.cme.msu.edu/) against the Silva 132 16S rRNA database using 80% confidence threshold. Microbiota data were analyzed using MicrobiomeAnalyst phyloseq-R package version 3.6.1 for different taxonomic levels (https://www.microbiomeanalyst.ca/MicrobiomeAnalyst/home.xhtml). Compositional data were analyzed using STAMP version 2.1.3 to identify differences in bacterial abundance across taxonomic levels.

### 2.7 Statistical analyses

Intention-to-treat analysis was performed using SPSS software version 20.0 (SPSS Inc, Chicago, USA). Considering the skewed distribution and non-parametric nature of our data, differences between groups were compared using the Mann-Whitney *U* test, while nominal data were compared using the Chi-square test. All tests were two-sided with *p* < 0.05 as considered statistically significant and data are presented as mean value ± standard error unless stated otherwise. It is important to note the statistical limitations of this study. The primary analyses focused on between-group comparisons at individual time points using non-parametric tests, which is a robust approach for evaluating the intervention effect at specific stages. However, we acknowledge that repeated-measures or mixed-effects models could offer additional insights into within-subject changes over time. Furthermore, due to the exploratory and hypothesis-generating nature of the subsidiary analyses on the numerous immune and microbiota variables, *p*-values were not adjusted for multiple comparisons. This approach is common in such studies to avoid overly conservative correction that might mask biologically relevant patterns. Therefore, the results from these specific analyses should be interpreted with caution, with emphasis placed on the overall coherence and biological plausibility of the findings across clinical, immunological, and microbial datasets, rather than on isolated *p*-values.

## 3 Results

### 3.1 Sociodemographic characteristics

**Figure 1:**
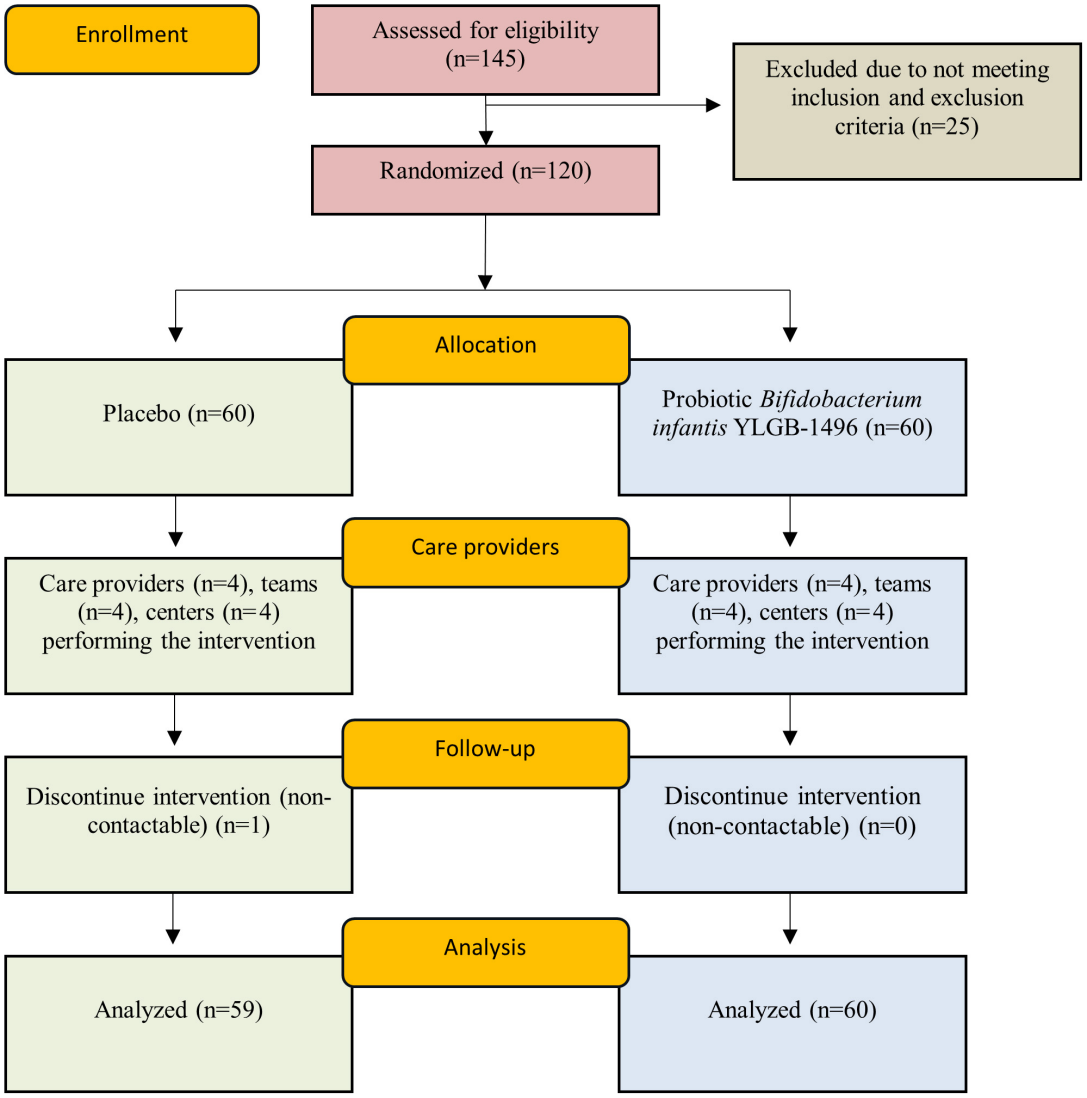
Consort flowchart detailing patients' recruitment, randomization and allocation. Healthy children (*n* = 119) randomly assigned to a double-blind administration with either placebo (*n* = 59) or probiotic *Bifidobacterium infantis* YLGB-1496 (*n* = 60).

145 subjects were screened for eligibility, only 120 fulfilled the inclusion and exclusion criteria. 120 subjects that were recruited, 1 subject dropped out during the 12-week period due being uncontactable, yielding 119 subjects after the 12-week study (Figure 1; probiotic *n* = 60; placebo *n* = 59). All 119 subjects provided fecal samples. No adverse effects were reported throughout the study and no subjects dropped out due to any complications. Insignificant differences were observed in all the general characteristics of probiotic and placebo subjects (Table 1). This indicates a comparable population; hence, health improvement or any observed effects within the two groups are more likely attributable to the intervention rather than pre-existing differences between participants.

**Table 1:** Baseline characteristics of healthy children (*n* = 119) randomly assigned to a double-blind administration with either placebo (*n* = 59) or probiotic *Bifidobacterium infantis* YLGB-1496 (*n* = 60).

**Baseline characteristics**	**Placebo**	**Probiotic *Bifidobacterium infantis* YLGB-1496**	*p*-value^*^
Sample size (*n*)	59	60	
**Gender, % (** ***n*** **)**			
Male	42% (26)	57% (36)	0.089^**^
Female	58% (36)	43% (27)	
Age (months)	25.73 ± 0.85	24.88 ± 0.80	0.474
Body weight (kg)	11.27 ± 0.33	11.43 ± 0.24	0.236
Height (cm)	84.53 ± 1.05	84.75 ± 1.57	0.338
BMI	15.77 ± 0.36	18.82 ± 2.51	0.634
Number of siblings	2.8 ± 0.19	3.17 ± 0.26	0.624
Smokers in family	0.32 ± 0.06	0.33 ± 0.06	0.896
Defecation frequency (per week)	7.31 ± 0.64	6.84 ± 0.44	0.916
History of food allergy	0.05 ± 0.03	0.13 ± 0.04	0.122
Hospitalization for the past 12-months	0.27 ± 0.08	0.45 ± 0.14	0.624
Incidence of RTI for the past 12 months	0.42 ± 0.11	0.49 ± 0.17	0.444
Incidence of diarrhea for the last 12 months	0.47 ± 0.12	0.54 ± 0.17	0.897
Antibiotic intake	0.07 ± 0.03	0.05 ± 0.03	0.681
Supplement intake	0.22 ± 0.05	0.3 ± 0.06	0.324
Dairy consumption	0.59 ± 0.06	0.57 ± 0.06	0.77
	% (*n*)	% (*n*)	*p*-value^**^
**Family income status**			
a) Low (< RM5,900)	64.52 (40)	68.25 (43)	0.658
b) > RM6,000	35.48 (22)	31.75 (20)	
**Number of people living together in a household**			
a) Less than or equal to 4 people	40.32 (25)	38.10 (24)	0.799
b) More than 4 people	59.68 (37)	61.90 (39)	
**Type of residence**			
a) Terrace/story/bungalow	72.58 (45)	63.49 (40)	0.276
b) Apartment	27.42 (17)	36.51 (23)	

^*^p-value obtained via Mann-Whitney U-test; ^**^p-value obtained via Chi-square test.

### 3.2 Questionnaires

#### 3.2.1 Respiratory health

The At baseline (week 0), no significant differences were observed between groups in respiratory outcomes, confirming comparable initial conditions (*p* > 0.6) for all parameters (Table 2). By week 6, participants in the probiotic group exhibited markedly fewer respiratory problems compared to placebo, with only 21.7% reporting issues vs. 45.8% in placebo (*p* = 0.005). This trend was sustained at week 12, where just 15.0% of probiotic subjects reported respiratory problems compared to 42.4% in placebo (*p* < 0.001). Clinical visits for respiratory problems were also significantly reduced in the probiotic group at both week 6 (*p* = 0.004) and week 12 (*p* = 0.003). Similarly, antibiotic use was substantially lower among probiotic recipients at week 6 (*p* = 0.007) and week 12 (*p* = 0.008).

**Table 2:** Subjects' responses for respiratory incidence questionnaire, (*n* = 119) randomly assigned to a double-blind administration with either placebo (*n* = 59) or probiotic *Bifidobacterium infantis* YLGB-1496 (*n* = 60).

**Week 0**				**Week 6**			**Week 12**		
**Parameters**	**Placebo**	**Probiotic** ***Bifidobacterium infantis*** **YLGB-1496**	***p*** **-value** ^*^	**Placebo**	**Probiotic** ***Bifidobacterium infantis*** **YLGB-1496**	***p*** **-value** ^*^	**Placebo**	**Probiotic** ***Bifidobacterium infantis*** **YLGB-1496**	***p*** **-value** ^*^
**#Past month respiratory problem**									
Yes *n* (%)	22 (37.3)	22 (36.7)	0.944^**^	27 (45.8)	13 (21.7)	0.005^**^	25 (42.4)	9 (15.0)	0.00^**^
No *n* (%)	37 (62.7)	38 (63.3)		32 (54.2)	47 (78.3)		34 (57.6)	51 (85.0)	
**#Clinical visit for respiratory problem in the past month**									
Yes *n* (%)	20 (33.9)	22 (36.7)	0.752^**^	24 (40.7)	10 (16.7)	0.004^**^	22 (37.3)	8 (13.3)	0.003^**^
No *n* (%)	39 (66.1)	38 (63.3)		35 (59.3)	50 (83.3)		37 (62.7)	52 (86.7)	
**#Antibiotic use for respiratory problem in the past month**									
Yes *n* (%)	20 (33.9)	18 (30)	0.648^**^	23 (39)	10 (16.7)	0.007^**^	20 (33.9)	8 (13.3)	0.008^**^
No *n* (%)	39 (66.1)	42 (70)		36 (61)	50 (83.3)		39 (66.1)	52 (86.7)	
**Growth parameters**									
Weight	11.21 ± 0.33	11.43 ± 0.24	0.18	11.69 ± 0.32	12.23 ± 0.25	0.09	12.56 ± 0.35	12.83 ± 0.27	0.42
Height	84.5 ± 1.06	85.8 ± 1.28	0.21	86.97 ± 0.94	90.56 ± 1.92	0.11	88.64 ± 1.03	91.8 ± 0.93	0.06
BMI	15.7 ± 0.35	16.97 ± 1.76	0.47	15.43 ± 0.3	15.35 ± 0.36	0.78	15.97 ± 0.35	15.27 ± 0.3	0.21
**Number of days with respiratory symptoms (per week) in the past month**									
Fever	0.58 ± 0.11	0.64 ± 0.16	0.86	0.79 ± 0.15	0.3 ± 0.08	0.01	0.83 ± 0.17	0.2 ± 0.07	0.00
Cough	0.76 ± 0.16	0.84 ± 0.19	0.99	1.17 ± 0.24	0.43 ± 0.12	0.00	1.06 ± 0.24	0.28 ± 0.1	0.00
Sneezing	0.66 ± 0.13	0.72 ± 0.17	0.86	1.13 ± 0.22	0.38 ± 0.1	0.00	1.02 ± 0.22	0.23 ± 0.07	0.00
Nose block	1.58 ± 0.28	1.64 ± 0.29	0.95	2.03 ± 0.3	0.92 ± 0.24	0.01	1.92 ± 0.31	0.63 ± 0.2	0.00
Wheezing	1.42 ± 0.27	1.38 ± 0.27	0.84	1.68 ± 0.29	0.83 ± 0.22	0.04	1.63 ± 0.28	0.63 ± 0.2	0.01
Sore throat	0.56 ± 0.11	0.45 ± 0.09	0.61	0.69 ± 0.13	0.28 ± 0.08	0.01	0.65 ± 0.13	0.2 ± 0.07	0.00
Runny nose	2.39 ± 0.42	2.39 ± 0.42	1.00	2.92 ± 0.44	1.24 ± 0.34	0.01	2.83 ± 0.44	0.96 ± 0.31	0.00
Poor appetite	0.56 ± 0.11	0.74 ± 0.17	0.66	0.79 ± 0.15	0.3 ± 0.08	0.01	0.93 ± 0.19	0.23 ± 0.07	0.00
Hoarseness	0.64 ± 0.13	0.5 ± 0.09	0.70	0.69 ± 0.13	0.25 ± 0.07	0.01	0.71 ± 0.15	0.2 ± 0.07	0.00
Body ache	0.47 ± 0.09	0.43 ± 0.09	0.75	0.66 ± 0.13	0.25 ± 0.07	0.01	0.48 ± 0.09	0.2 ± 0.07	0.01
Fatigue	0.08 ± 0.08	0.12 ± 0.12	1.00	0.18 ± 0.11	0.03 ± 0.03	0.30	0.17 ± 0.12	0.03 ± 0.03	0.30
Vomiting	0 ± 0	0.03 ± 0.03	0.32	0.2 ± 0.11	0.03 ± 0.03	0.16	0.05 ± 0.04	0.03 ± 0.03	0.55
Headache	0.46 ± 0.09	0.43 ± 0.09	0.80	0.66 ± 0.13	0.28 ± 0.08	0.02	0.51 ± 0.09	0.23 ± 0.07	0.02
Thick mucus	0.69 ± 0.15	0.67 ± 0.16	0.90	0.93 ± 0.19	0.33 ± 0.1	0.00	0.86 ± 0.18	0.2 ± 0.07	0.00
Pain swallowing	0.1 ± 0.08	0.03 ± 0.03	0.54	0.18 ± 0.11	0.00 ± 0.00	0.08	0.15 ± 0.11	0.00 ± 0.00	0.15

^*^p-value obtained via Mann-Whitney U-test; ^**^p-value obtained via Chi-square test.

This study demonstrates that daily supplementation with *B. infantis* YLGB-1496 significantly reduced the incidence of respiratory tract illness episodes (RTIs), associated symptoms, and the need for medical intervention compared to placebo over a 12-week period. The consistent reductions in clinical visits (week 6: *p* = 0.004; week 12: *p* = 0.003) and antibiotic use (week 6: *p* = 0.007; week 12: *p* = 0.008) highlight the clinical relevance of probiotic intervention in lowering healthcare burden.

The protective effect of *B. infantis* YLGB-1496 against respiratory illness was evident by the observed reductions in key respiratory symptoms, including fever, cough, sneezing, and nasal obstruction, further emphasize the protective effect of *B. infantis* YLGB-1496. At week 12, the probiotic group had markedly fewer days with cough (*p* < 0.001) and sore throat (*p* < 0.001), supporting symptomatic relief alongside reduced infection frequency.

#### 3.2.2 Gastrointestinal health

At baseline (week 0), gastrointestinal outcomes were comparable between groups (*p* > 0.400) across measures (Table 3). By week 6, probiotic supplementation significantly improved bowel regularity, with participants in the probiotic group reporting a higher defecation frequency per week, (*p* < 0.001). This effect remained consistent at week 12 (*p* = 0.001). Crucially, this change was accompanied by a significant reduction in the incidence of diarrhea (Table 3), indicating that the increased frequency represented a normalization of bowel habits and an improvement in overall gastrointestinal comfort, rather than a laxative effect or diarrhea. Importantly, the occurrence of diarrhea in the past month was significantly lower in the probiotic group at week 6 (*p* = 0.002), indicating a protective effect against gastrointestinal infections. This shift likely reflects a resolution of mild, sub-clinical constipation common in preschool children.

**Table 3:** Subjects' responses for gastrointestinal incidence questionnaire, (*n* = 119) randomly assigned to a double-blind administration with either placebo (*n* = 59) or probiotic *Bifidobacterium infantis* YLGB-1496 (*n* = 60).

**Week 0**				**Week 6**			**Week 12**		
**Parameters**	**Placebo**	**Probiotic** ***Bifidobacterium infantis*** **YLGB-1496**	***p*** **-value** ^*^	**Placebo**	**Probiotic** ***Bifidobacterium infantis*** **YLGB-1496**	***p*** **-value** ^*^	**Placebo**	**Probiotic** ***Bifidobacterium infantis*** **YLGB-1496**	***p*** **-value** ^*^
Period (days) for stomach discomfort (such as flatulence and bloating) per week	0.29 ± 0.11	0.35 ± 0.12	0.80	0.32 ± 0.13	0.31 ± 0.14	0.60	0.46 ± 0.16	0.29 ± 0.09	0.718
Period (days) for stomach ache per week	0.17 ± 0.08	0.28 ± 0.12	0.74	0.15 ± 0.06	0.24 ± 0.09	0.57	0.33 ± 0.12	0.33 ± 0.11	0.682
Defecation times per week	7.71 ± 0.69	7.58 ± 0.63	0.76	7.6 ± 0.62	12.76 ± 0.84	0.00	7.9 ± 0.75	11.61 ± 0.85	0.001
**#Occurrence of diarrhea in the past month**									
Yes (*n*, %)	28 (47.46)	32 (53.33)	0.522^**^	26 (44.07)	11 (18.33)	0.002^**^	19 (32.20)	13 (21.67)	0.195^**^
No (*n*, %)	31 (52.54)	28 (46.67)		33 (55.93)	49 (81.67)		40 (67.80)	47 (78.33)	
Past month diarrhea incident (number of times per child)	0.83 ± 0.14	0.98 ± 0.14	0.39	0.85 ± 0.13	0.52 ± 0.15	0.01	0.63 ± 0.13	0.48 ± 0.14	0.252
Past month clinical visit due to diarrhea incident (number of times per child)	0.53 ± 0.09	0.55 ± 0.07	0.54	0.49 ± 0.09	0.23 ± 0.08	0.00	0.37 ± 0.09	0.28 ± 0.08	0.291
**Number of days with gastrointestinal symptoms (per week) in the past month**									
Fever	1.15 ± 0.23	1.25 ± 0.23	0.80	1.52 ± 0.27	0.08 ± 0.05	0.00	1 ± 0.24	0.25 ± 0.13	0.012
Vomit	1.07 ± 0.23	1.2 ± 0.23	0.65	1.52 ± 0.27	0.12 ± 0.06	0.00	0.93 ± 0.24	0.15 ± 0.06	0.039
Dysentery	0.03 ± 0.02	0.03 ± 0.02	0.99	0.03 ± 0.02	0.02 ± 0.02	0.55	0.02 ± 0.02	0.03 ± 0.02	0.57
Stomach ache	1.21 ± 0.23	1.32 ± 0.23	0.63	1.65 ± 0.29	0.26 ± 0.1	0.00	1.12 ± 0.26	0.3 ± 0.1	0.048
Nausea	0.03 ± 0.02	0.1 ± 0.05	0.40	0.07 ± 0.04	0.14 ± 0.09	0.99	0.03 ± 0.02	0.07 ± 0.04	0.652
Poor appetite	1.27 ± 0.25	1.3 ± 0.25	1.00	1.55 ± 0.27	0.24 ± 0.1	0.00	1.14 ± 0.26	0.48 ± 0.19	0.067
Fatigue	0.05 ± 0.03	0.17 ± 0.09	0.47	0.08 ± 0.06	0.16 ± 0.09	0.70	0.17 ± 0.12	0.23 ± 0.09	0.204
Dizziness	0.03 ± 0.02	0.03 ± 0.02	0.99	0.05 ± 0.03	0.08 ± 0.05	1.00	0.03 ± 0.02	0.08 ± 0.04	0.408
Headache	0.03 ± 0.02	0.03 ± 0.02	0.99	0.07 ± 0.04	0.08 ± 0.05	0.99	0.03 ± 0.02	0.08 ± 0.04	0.408
Dehydration	1.05 ± 0.23	1.2 ± 0.23	0.66	1.53 ± 0.27	0.14 ± 0.09	0.00	0.98 ± 0.24	0.2 ± 0.09	0.015
Rectal discomfort	0.17 ± 0.08	0.03 ± 0.02	0.13	0.07 ± 0.04	0.08 ± 0.04	0.72	0.15 ± 0.12	0.13 ± 0.06	0.485
Past month diarrhea incident (number of days)	1.19 ± 0.18	1.37 ± 0.19	0.47	1.14 ± 0.18	0.45 ± 0.14	0.00	0.81 ± 0.17	0.45 ± 0.12	0.156
Past month diarrhea incident (frequency per day)	1.29 ± 0.19	1.55 ± 0.21	0.31	1.25 ± 0.19	0.53 ± 0.16	0.01	0.88 ± 0.17	0.58 ± 0.15	0.235

^*^p-value obtained via Mann-Whitney U-test; ^**^p-value obtained via Chi-square test.

Symptom-specific improvements were also observed. At week 6, the probiotic group experienced fewer days with fever (*p* < 0.001), vomiting (*p* < 0.001), stomach ache (*p* < 0.001), poor appetite (*p* < 0.001), and dehydration (*p* < 0.001) compared with placebo. These benefits persisted through week 12, where probiotic recipients continued to report lower frequencies of fever (*p* = 0.012), vomiting (*p* = 0.039), stomach ache (*p* = 0.048), and dehydration (*p* = 0.015). Clinical visits for diarrhea were also significantly reduced in the probiotic group at week 6 (*p* < 0.001).

The present findings indicate that *B. infantis* YLGB-1496 supplementation provides significant gastrointestinal benefits, particularly in reducing diarrhea incidence and associated symptoms. By week 6, probiotic administration reduced the proportion of children experiencing diarrhea to 18.3% compared with 44.1% in the placebo group (*p* = 0.002).

### 3.3 Immunological and biochemical proteins profile

**Figure 2:**
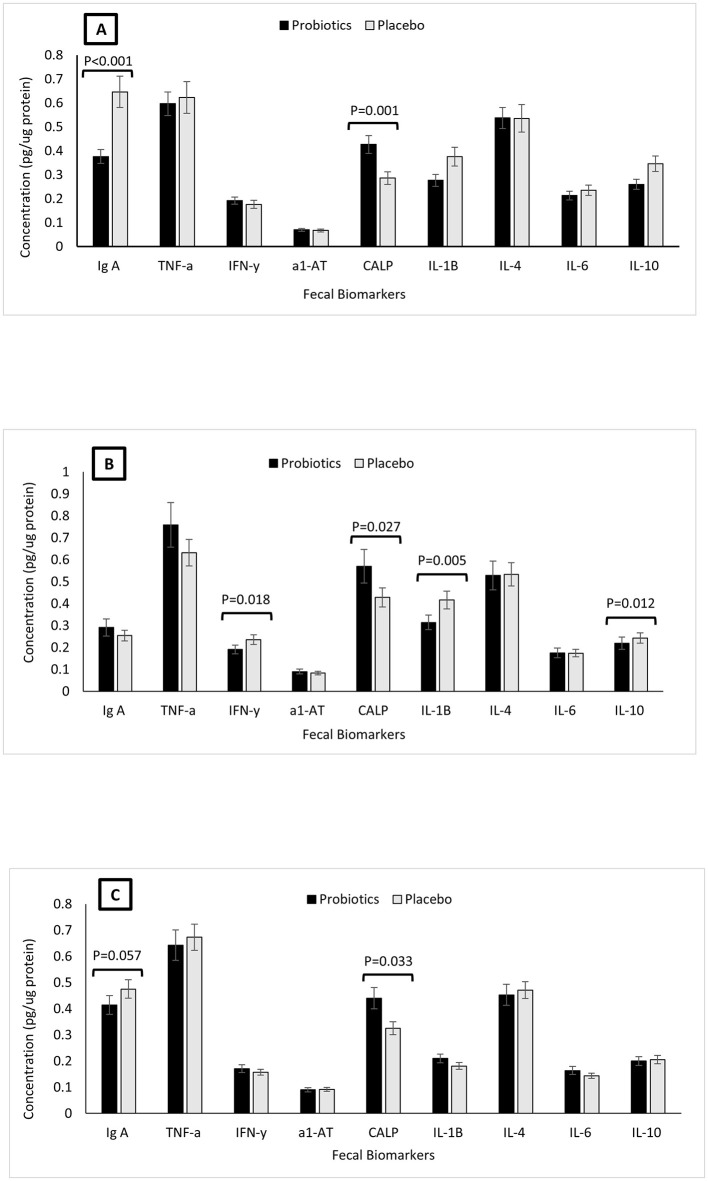
Concentrations of fecal biomarkers as obtained from fecal samples of children (*n* = 119) randomly assigned to a double-blind administration of 12 weeks with either placebo (*n* = 59; gray) or probiotic *Bifidobacterium infantis YLGB-1496* (*n* = 60; black). Results are expressed as means; error bars (SEM). **(A)** Comparison of biomarkers between probiotic and placebo taken at week 0. **(B)** Comparison of biomarkers between probiotic and placebo taken at week 6. **(C)** Comparison of biomarkers between probiotic and placebo taken at week 12.

The impact of *B. infantis* YLGB-1496 on fecal biomarkers of gut inflammation and immunity is shown in Figure 2. At baseline (Week 0), the probiotic and placebo groups were broadly comparable for most fecal biomarkers, including TNF-α, IFN-γ, IL-4, IL-6, IL-10, and α1-antitrypsin (all *p* > 0.05; Figure 2A). However, two significant baseline differences were noted: the probiotic group had a lower concentration of fecal IgA (*p* = 0.0002) and a higher concentration of fecal calprotectin (*p* = 0.0006) compared to the placebo group, setting a conservative starting point for assessing probiotic efficacy.

By Week 6, supplementation induced a clear anti-inflammatory shift in the gut (Figure 2B). The probiotic group exhibited significant reductions in the pro-inflammatory cytokines IFN-γ (*p* = 0.018) and IL-1β (*p* = 0.005), alongside a marked decrease in the inflammation marker calprotectin (*p* = 0.027) relative to the placebo. The regulatory cytokine IL-10 was also relatively preserved in the probiotic group (*p* = 0.012). In contrast, levels of TNF-α, IL-6, and IL-4 remained stable and comparable between groups (all *p* > 0.05), indicating a selective immunomodulatory effect rather than global immunosuppression.

At Week 12, the beneficial effects on the gut environment were sustained (Figure 2C). The reduction in fecal calprotectin remained significant (*p* = 0.033). Furthermore, fecal IgA, which was initially lower in the probiotic group, showed a strong trend toward increase relative to the placebo (*p* = 0.057), suggesting a bolstering of mucosal immunity over the intervention period. The differences in IFN-γ and IL-1β observed at Week 6 were attenuated and no longer significant (*p* = 0.865 and *p* = 0.278, respectively). Levels of TNF-α, IL-6, and IL-4 remained consistently unchanged throughout the study (all *p* > 0.05).

**Figure 3:**
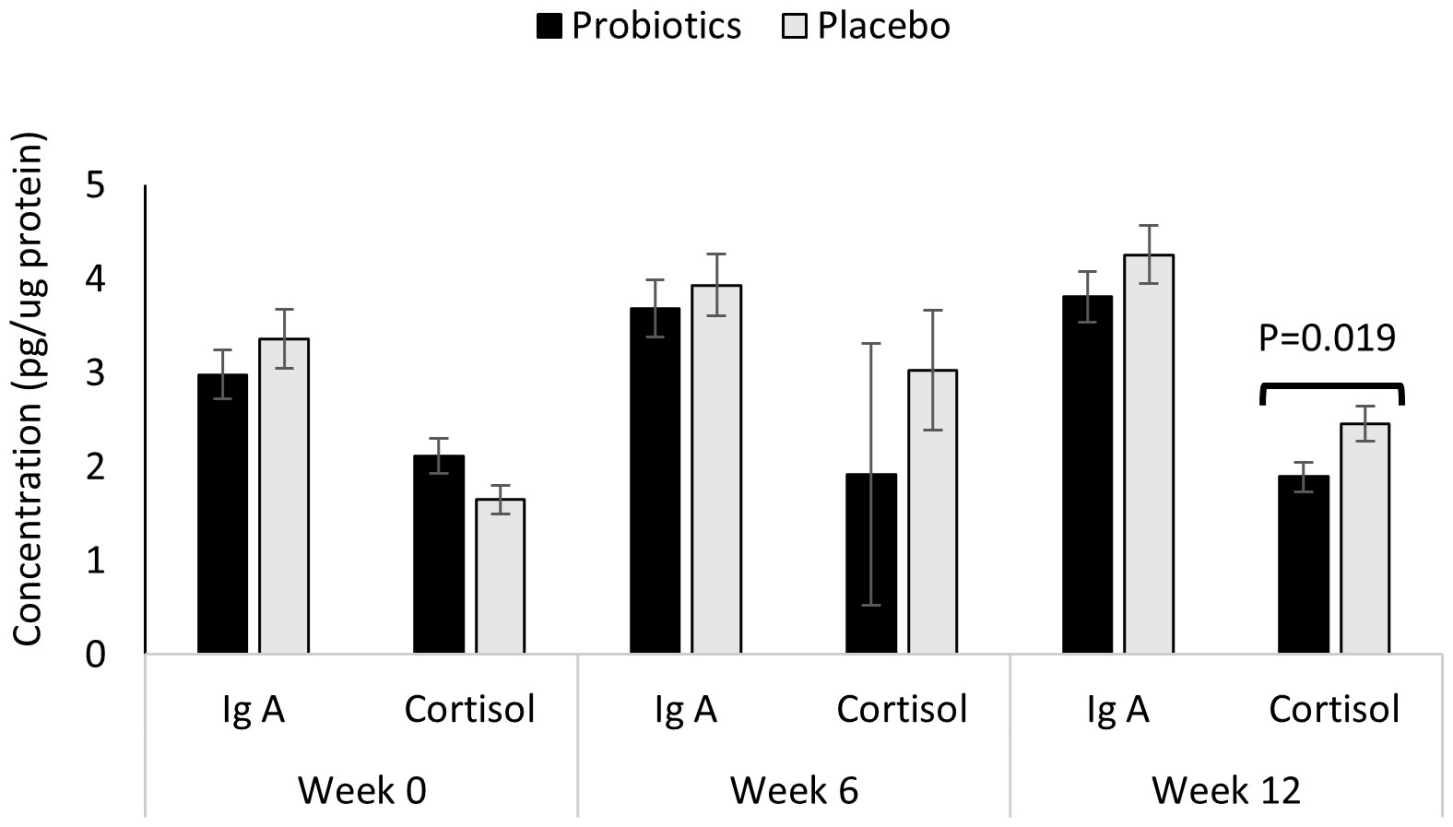
Concentrations of immune and stress biomarkers as obtained from oral samples of children (*n* = 119) randomly assigned to a double-blind administration of 12 weeks with either placebo (*n* = 59; gray) or probiotic *Bifidobacterium infantis YLGB-1496* (*n* = 60; black). Results are expressed as means; error bars (SEM). Comparison of biomarkers between probiotic and placebo taken at week 0, week 6 and week 12, respectively.

Oral biomarkers, reflecting systemic immune and stress responses, are presented in Figure 3. At baseline, no significant differences were observed between groups for salivary IgA or cortisol (all *p* > 0.05). These levels remained comparable at Week 6.

By Week 12, however, a significant divergence was observed in salivary cortisol (*p* = 0.019), which was reduced in the probiotic group compared to the placebo (Figure 3). As cortisol is the primary effector of the hypothalamic-pituitary-adrenal (HPA) axis, (22, 23) this reduction suggests a potential systemic effect of probiotic supplementation on stress physiology. In contrast, salivary IgA levels did not show significant differences between the groups at any time point (*p* > 0.05).

### 3.4 Gut microbiota profiling

#### 3.4.1 Alpha diversity

**Figure 4:**
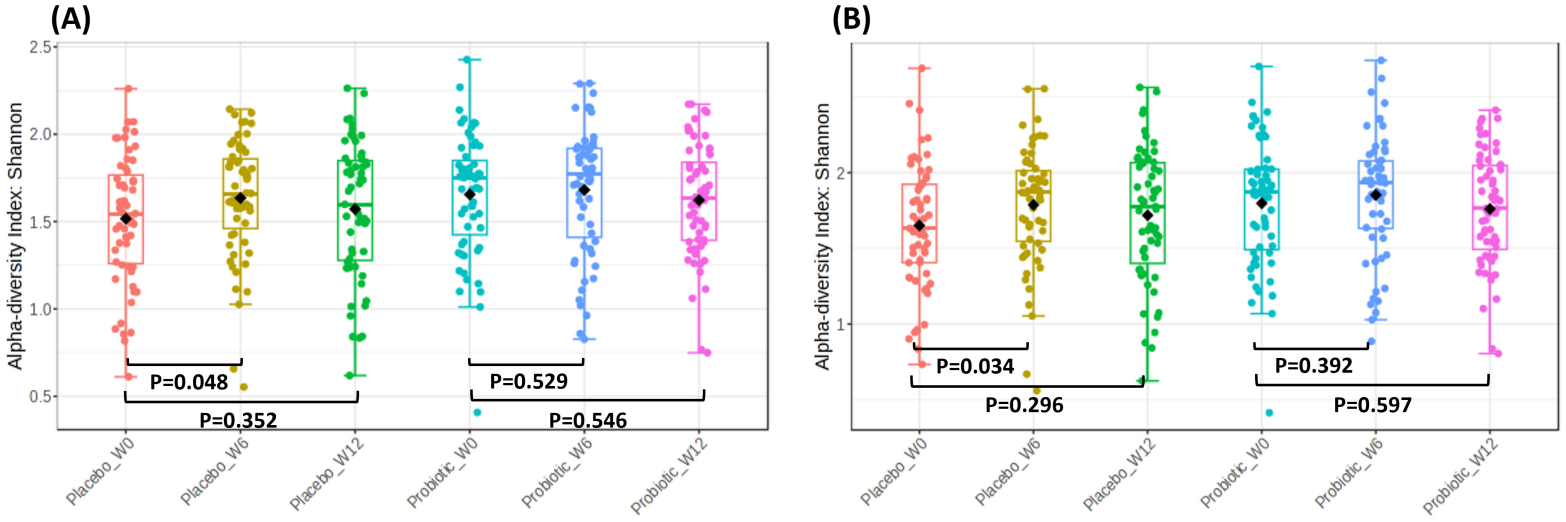
Alpha diversity plots for fecal microbiota at baseline (week-0), week-6 and end of study (week-12), upon administration of probiotic *Bifidobacterium infantis* YLGB-1496 or placebo. Differences in diversity within group as measured by the Shannon index for **(A)** order and **(B)** family. The line inside the box represents the median, whereas the whiskers represent the lowest and highest values within the interquartile range. Outliers, as well as individual sample values, are shown as dots. Statistical significance was analyzed using the Mann–Whitney *U* test. *n* = 119 (probiotic *n* = 60 and placebo *n* = 59).

Alpha diversity measures differences within samples via the use of several indices. The Shannon index measures evenness, to determine differences in abundances of the operational taxonomic unit (OTU) within the same sample. Higher Shannon values indicate higher evenness. Shannon index, was compared within groups across different timepoints at the order and family levels (Figure 4). At the order level (Figure 4A), the placebo group demonstrated a significant increase in Shannon diversity between week 0 and week 6 (*p* = 0.048), indicating a temporary rise in microbial richness and/or evenness. However, this effect was not sustained, as diversity returned to baseline by week 12 (*p* = 0.352). In contrast, the probiotic group showed no significant changes in Shannon diversity at either week 6 (*p* = 0.529) or week 12 (*p* = 0.546) compared with baseline.

Similarly, at the family level (Figure 4B), the placebo group exhibited a transient increase in Shannon diversity from week 0 to week 6 (*p* = 0.034), reflecting a short-term enhancement in richness and/or evenness. This increase was not maintained at week 12 (*p* = 0.296). The probiotic group again showed no significant changes at week 6 (*p* = 0.392) or week 12 (*p* = 0.597) relative to baseline.

**Figure 5:**
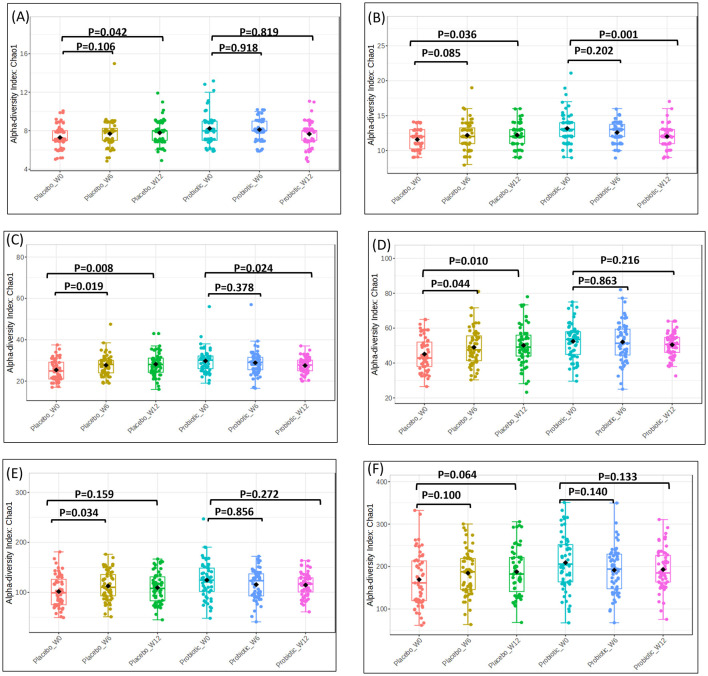
Alpha diversity plots for fecal microbiota at baseline (week-0), week-6 and end of study (week-12), upon administration of probiotic *Bifidobacterium infantis* YLGB-1496 or placebo. Differences in diversity within group as measured by the Chao1 index for **(A)** phylum, **(B)** class, **(C)** order, **(D)** family, **(E)** genus and **(F)** species. The line inside the box represents the median, whereas the whiskers represent the lowest and highest values within the interquartile range. Outliers, as well as individual sample values, are shown as dots. Statistical significance was analyzed using the Mann–Whitney *U* test. *n* = 119 (probiotic *n* = 60 and placebo *n* = 59).

Meanwhile, the Chao1 index to evaluate microbial richness across six taxonomic levels (Figure 5). At the (Figure 5A) phylum and (Figure 5B) class levels, the placebo group exhibited a significant increase in Chao1 richness at week 6 compared with baseline (*p* < 0.05), which was not maintained at week 12 (*p* > 0.05). At the (Figure 5C) order and (Figure 5D) family levels, the placebo group similarly showed a transient increase in richness at week 6 (order: *p* < 0.05; family: *p* < 0.05) that returned to baseline by week 12. The (Figure 5E) genus and (Figure 5F) species levels also showed a significant, but short-lived, rise in richness in the placebo group at week 6 (*p* < 0.05) with no persistent change at week 12. By contrast, the probiotic group did not show significant changes at week 6 at most taxonomic levels; however, a decrease in Chao1 richness was observed in the probiotic group at the class and order levels at week 12 (*p* < 0.05), while family, genus and species richness remained statistically unchanged at both week 6 and week 12 (*p* > 0.05).

#### 3.4.2 Beta diversity

**Figure 6:**
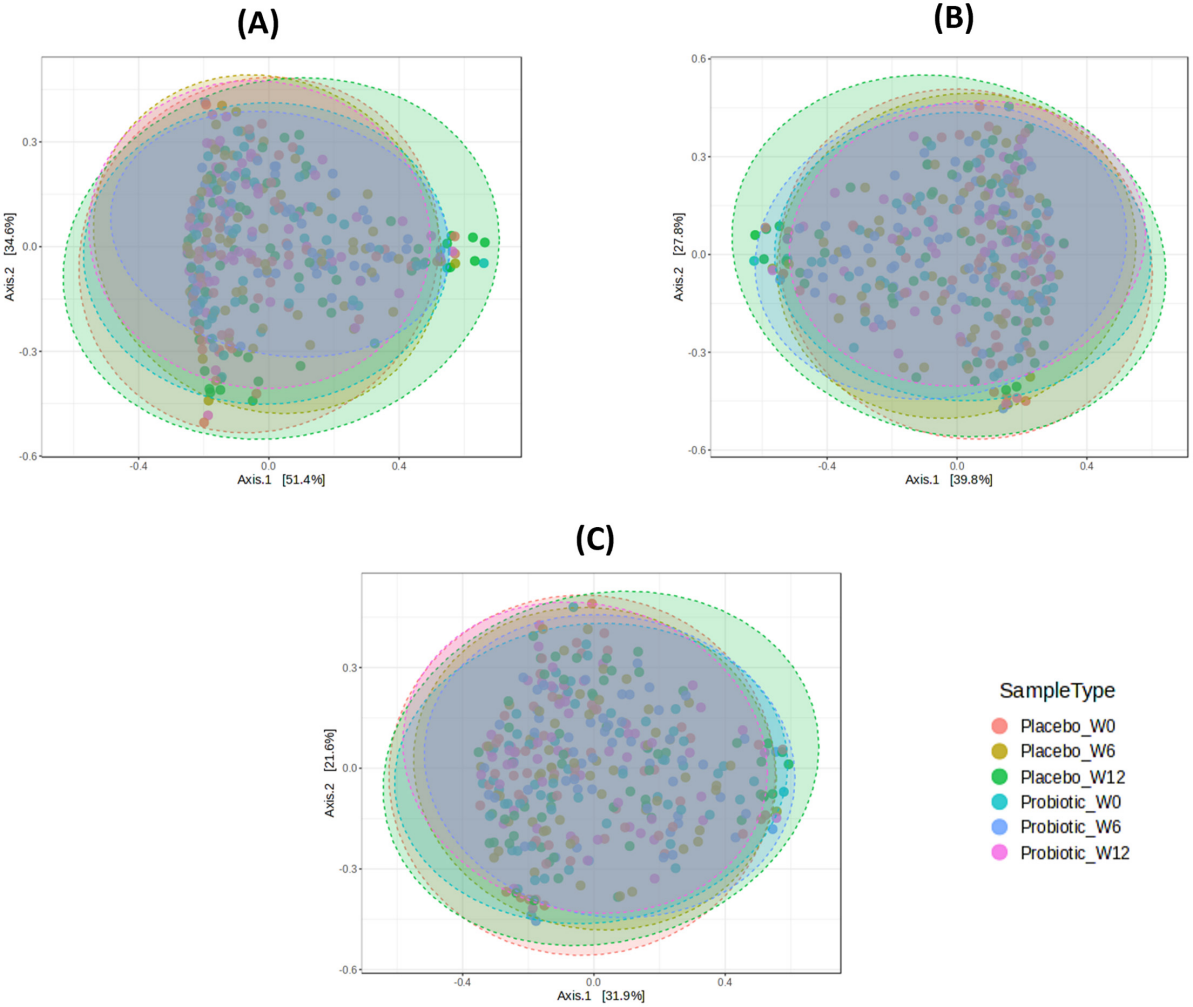
Beta diversity as measured by the Bray-Curtis dissimilarity index are plotted at baseline (week-0) and after week-12 (W12), upon administration of probiotic *Bifidobacterium infantis* YLGB-1496 or placebo. Principal coordinates analysis (PCoA) as measured by PERMANOVA. At week 12, marginal differences were observed at the **(A)** phylum (*p* = 0.056), **(B)** class (*p* = 0.062), and **(C)** order (*p* = 0.099) levels. *n* = 119 (probiotic *n* = 60 and placebo *n* = 59).

Beta diversity of gut microbial communities was assessed using the Bray-Curtis dissimilarity index and visualized by principal coordinates analysis (PCoA). Differences between participants receiving probiotic *B. infantis* YLGB-1496 and placebo were evaluated by permutational multivariate analysis of variance (PERMANOVA) across multiple taxonomic levels (Figure 6).

At week 12, marginal differences in microbial community composition were observed at higher taxonomic ranks, including the (Figure 6A) phylum (*p* =0.056), (Figure 6B) class (*p* =0.062), and (Figure 6C) order (*p* =0.099) levels. No significant differences were detected at the family, genus, or species levels. These results indicate that probiotic supplementation did not produce significant restructuring of the gut microbiota, although trends toward divergence were evident at broader taxonomic levels.

#### 3.4.3 Compositional differences

Analysis of taxonomic shifts via STAMP revealed distinct ecological trajectories between the probiotic and placebo groups over the 12-week intervention, illustrating a modulatory effect of *B. infantis* YLGB-1496 on the gut ecosystem of healthy young children.

**Figure 7:**
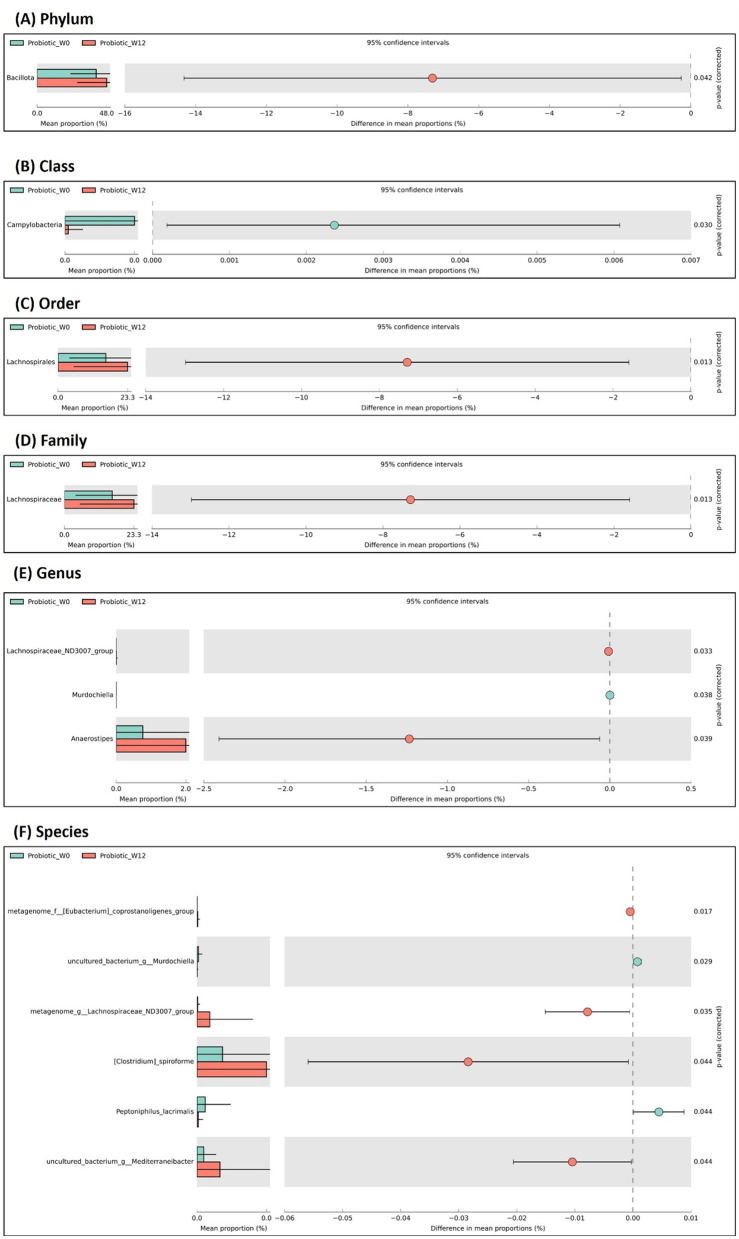
Extended bar plot in STAMP, illustrating the significant fecal bacteria abundance across taxonomy plotted at baseline (week-0) and after week-12 upon administration of probiotic *Bifidobacterium infantis* YLGB-1496. Comparison of gut microbiota at level of **(A)** phylum, **(B)** class, **(C)** order, **(D)** family, **(E)** genus, **(F)** species, respectively. *n* = 119 (probiotic *n* = 60 and placebo *n* = 59).

In the probiotic group, supplementation was associated with a reduction in the relative abundance of several fermentative, butyrate-producing taxa, including the genera Lachnospiraceae ND3007 group, Anaerostipes, and Mediterraneibacter (Figure 7; *p* < 0.05). While these taxa are generally considered beneficial, their relative decrease may indicate a probiotic-driven ecological succession (24–26), where the introduced *B. infantis* competes for dietary substrates, leading to a rebalancing of the microbial network. This is supported by a significant increase in the class Campylobacteria (*p* < 0.05), which includes members known for utilizing host-derived mucins. The concurrent rise in the genus Murdochiella and the species *Peptoniiphilus lacrimalis* (*p* < 0.05), both part of the core infant microbiota (27), further suggests that probiotic administration supported the maturation of a stable, host-adapted microbial community rather than a simple proliferation of all beneficial genera (28).

**Figure 8:**
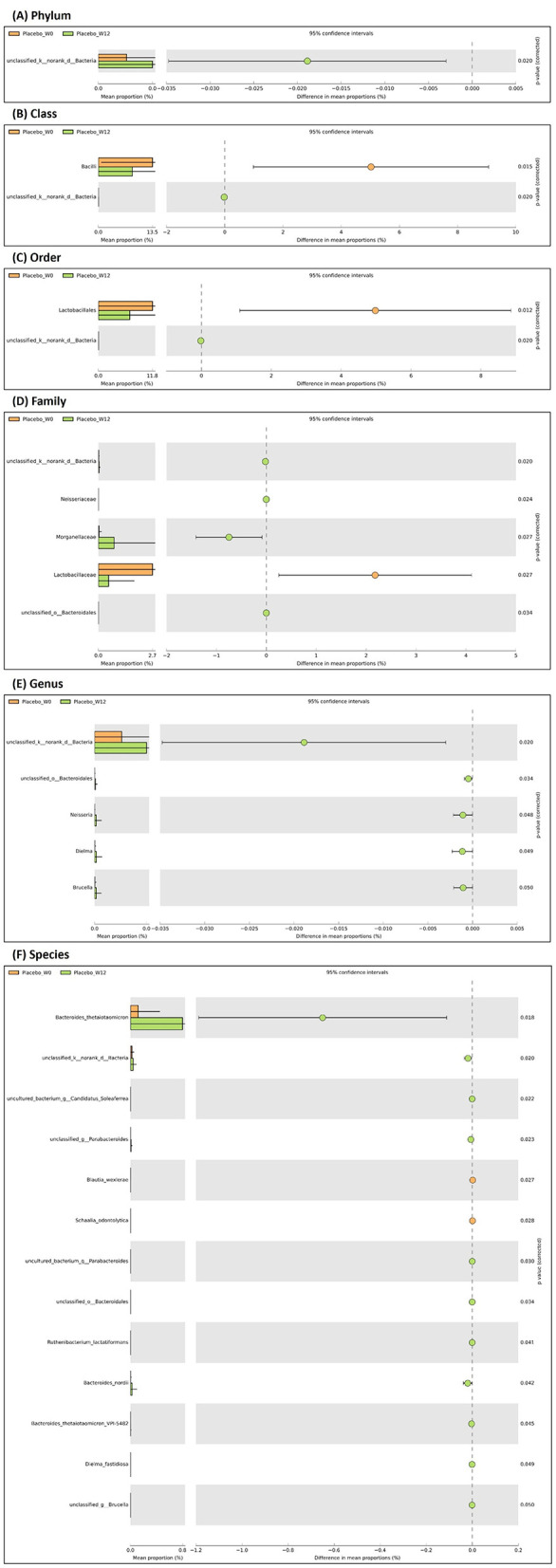
Extended bar plot in STAMP, illustrating the significant fecal bacteria abundance across taxonomy plotted at baseline (week-0) and after week-12 in placebo group. Comparison of gut microbiota at level of **(A)** phylum, **(B)** class, **(C)** order, **(D)** family, **(E)** genus, **(F)** species, respectively. *n* = 119 (probiotic *n* = 60 and placebo *n* = 59).

In stark contrast, the placebo group exhibited a trajectory consistent with less favorable ecological shifts. A marked and significant increase in the class Bacilli, order Lactobacillales, and family Lactobacillaceae was observed (Figure 8; *p* < 0.05). While Lactobacillaceae are typically viewed as beneficial, their pronounced expansion in the absence of probiotic intervention may signal an imbalance, potentially reflecting a less diverse or more unstable gut environment (29). More critically, the placebo group demonstrated a significant decline in several taxa crucial for gut health. This included a reduction in the family Neisseriaceae and the genus Neisseria (*p* < 0.05), which are early colonizers involved in initial immune education (30). Furthermore, a significant loss was observed in key butyrate-producing and mucin-degrading species, such as *Bacteroides thetaiotaomicron, Bacteroides nordii, Candidatus Soleaferrea*, and *Dielma fastidiosa* (*p* < 0.05). The depletion of these species is particularly noteworthy, as *B. thetaiotaomicron* is a champion polysaccharide degrader that supports overall microbial metabolism (31), and *Candidatus Soleaferrea* and Dielma species are important for producing the short-chain fatty acid butyrate, which is vital for colonocyte health and anti-inflammatory signaling (32, 33). The expansion of *Blautia wexlerae* and *Schaalia odontolytica* in the placebo group (*p* < 0.05) may represent a compensatory shift, but one that is associated with a less optimal, post-weaning microbial profile (34).

Collectively, these divergent patterns underscore the stabilizing influence of *B. infantis* YLGB-1496. The probiotic group maintained a more stable and mature community structure, while the placebo group drifted toward a configuration characterized by the loss of critical commensals involved in immune modulation and barrier integrity. This suggests that probiotic supplementation in healthy infants does not merely add a single strain but actively guides the developing microbiota toward a resilient and health-associated state, protecting against the erosion of beneficial functions observed in the untreated group.

## 4 Discussion

This study demonstrates that daily supplementation with *B. infantis* YLGB-1496 confers broad benefits in healthy toddlers, spanning clinical outcomes, immune regulation, and gut microbial ecology. Clinically, children in the probiotic group experienced fewer respiratory tract illness episodes, gastrointestinal disturbances, and healthcare visits compared with placebo. The reductions in cough, sore throat, diarrhea, fever, vomiting, and dehydration highlight both systemic and gut-specific protective effects. Importantly, the probiotic also reduced antibiotic use, a clinically relevant outcome given the growing burden of antimicrobial resistance. These results align with prior reports showing that bifidobacteria and lactobacilli strains can reduce infectious illness, shorten illness duration, and decrease medication reliance in children (9, 35–37).

The immunological data from this study provide compelling evidence that *B. infantis* YLGB-1496 supplementation elicits a targeted immunomodulatory effect in healthy children, characterized by the selective fine-tuning of inflammatory pathways rather than a broad immunosuppressive action. This is a hallmark of a proficient probiotic strain that can enhance defense mechanisms without compromising basal immunity.

Our findings demonstrate a clear anti-inflammatory profile in the gut by week 6, marked by significant reductions in the pro-inflammatory cytokines IL-1β and IFN-γ, alongside a decrease in fecal calprotectin, a robust clinical marker of neutrophilic intestinal inflammation. This coordinated downregulation of key inflammatory mediators indicates a successful attenuation of underlying immune activity. Crucially, this occurred concurrently with the relative preservation of IL-10, a pivotal regulatory cytokine essential for maintaining mucosal tolerance and preventing excessive inflammation (38). This pattern suppressing pro-inflammatory signals while sustaining regulatory ones is consistent with an ideal probiotic response, promoting immune homeostasis rather than blanket suppression (39). The stability of TNF-α, IL-6, and IL-4 levels throughout the study further underscores this point, confirming that fundamental immune surveillance and Th2-related responses remained intact (40).

Beyond the local gut effects, the trajectory of fecal IgA and the systemic measurement of salivary cortisol offer insights into broader physiological benefits. The observed trend toward increased fecal IgA at week 12 suggests a reinforcement of the mucosal barrier, a primary defense mechanism against pathogens. Probiotics are known to enhance secretory IgA production, which is critical for immune exclusion and maintaining host-commensal mutualism (41). More notably, the significant reduction in salivary cortisol levels in the probiotic group points to a potential systemic effect extending to the gut-brain axis. The hypothalamic-pituitary-adrenal (HPA) axis is a key mediator of the stress response, and its dysregulation is linked to both intestinal permeability and inflammation (23). Our finding aligns with a growing body of evidence suggesting that specific probiotic strains can modulate the HPA axis, potentially by reducing systemic inflammatory tone or through the production of neuroactive metabolites (22, 42). The concurrent dampening of intestinal inflammation (reduced calprotectin, IL-1β) and systemic stress marker (cortisol) presents a coherent picture of improved systemic homeostasis, plausibly driven by the probiotic-induced stabilization of the gut ecosystem.

Collectively, these immunological findings are consistent with the established mechanisms of proficient probiotic strains, which often involve the induction of regulatory T cells, enhancement of epithelial barrier function, and a resultant balanced cytokine milieu (43, 44). The data for *B. infantis* YLGB-1496 firmly support its role as an effective nutritional strategy for promoting a balanced immune response and overall resilience in the developing pediatric system.

Alpha diversity analyses revealed distinct temporal dynamics. In the placebo group, microbial richness and evenness transiently increased at week 6 but returned to baseline by week 12, reflecting environmentally driven or stochastic fluctuations commonly observed in early-life microbiota (45, 46). In contrast, the probiotic group maintained stable diversity across most taxonomic levels, with a modest reduction in richness at higher levels (class, order) by week 12. This stability suggests that *B. infantis* YLGB-1496 supports ecological resilience rather than driving wholesale compositional shifts, consistent with the concept that resilient microbiota rather than maximally diverse microbiota is associated with health (47, 48).

Beta diversity analysis revealed probiotic supplementation with *B. infantis* YLGB-1496 did not result in significant alterations in gut microbial community structure, although marginal differences were observed at higher taxonomic levels. These findings are in line with previous studies showing that probiotics often do not produce major changes in beta diversity, particularly in healthy adults where the gut microbiota is stable and resilient to perturbation (49, 50).

Rather than driving broad compositional restructuring, probiotics may act through subtle ecological modulation or by influencing functional and metabolic pathways. Prior research suggests that probiotic effects are more evident at the level of host-microbiome interactions, such as immune signaling or metabolite production, rather than through large shifts in community composition (51). Our results therefore highlight the importance of complementing diversity measures with functional analyses to fully capture the impact of probiotic interventions.

Taxonomic analysis reveals that *B. infantis* YLGB-1496 functions as an ecological stabilizer, guiding the gut microbiota of healthy children toward a more mature and resilient state. A key finding was a reduction in certain butyrate-producing genera such as Lachnospiraceae ND3007 group, which likely reflects a probiotic-driven ecological succession. The successful colonization of *B. infantis* YLGB-1496 appears to have competitively reshaped metabolic niches, favoring the rise of stable, host-adapted taxa like Murdochiella and mucin-utilizing Campylobacteria, signifying a progression toward a more advanced gut ecosystem.

In contrast, the placebo group drifted toward a less favorable state. This was marked not only by the expansion of Lactobacillaceae potentially indicating a lower-diversity, unstable environment but, more critically, by a significant erosion of foundational commensals. The loss of immune-priming Neisseria, the master polysaccharide degrader *Bacteroides thetaiotaomicron*, and key butyrate producers like *Candidatus Soleaferrea* compromised the community's metabolic and immunomodulatory capacity.

Collectively, these divergent trajectories provide a mechanistic basis for the observed clinical benefits. By preventing the functional impoverishment seen in the placebo group, *B. infantis* YLGB-1496 directly supported the enhanced mucosal immunity and reduced inflammation we documented, underscoring its role in proactively stabilizing the developing gut ecosystem against common perturbations.

A recognized consideration in our microbiota analysis is that 16S rRNA gene sequencing, as employed in this study, does not distinguish between DNA derived from viable and non-viable cells. Therefore, the taxonomic profiles represent the total microbial biomass (living and dead) present in the fecal samples. However, this approach is well-suited for assessing overall community-level changes and ecological stability, which was the primary goal of our microbiota analysis (52). The observed preservation of beneficial SCFA-producing genera in the probiotic group, as compared to the drift seen in the placebo group, reflects a meaningful difference in the gut ecosystem structure attributable to the intervention.

It is important to acknowledge the limitations of this study. The assessment of respiratory and gastrointestinal symptoms relied on parent-reported questionnaires, which, while practical and validated for community-based trials, are subject to potential recall and reporting bias in the absence of clinical confirmation. Furthermore, we did not perform a direct comparative analysis of Bifidobacterium genus abundance, which would have more conclusively demonstrated colonization by the administered probiotic. Despite these limitations, the consistency of the parent-reported symptoms with the objective reductions in clinical visits and antibiotic prescriptions strengthens the validity of the clinical benefits. Moreover, the primary focus of this study was on the functional, clinical, and broader ecological outcomes of the intervention, all of which were successfully demonstrated. Future studies may incorporate clinician-confirmed diagnoses and utilize strain-specific techniques, such as metagenomic sequencing or quantitative PCR, to definitively track the probiotic strain and further solidify the causal link between its colonization and the observed health benefits.

Taken together, these findings highlight the multifaceted benefits of *B. infantis* YLGB-1496. Clinically, supplementation reduced infection-related morbidity and healthcare burden. Immunologically, it tempered excessive inflammation while supporting regulatory and barrier functions. Ecologically, it stabilized the microbiome, preserved functional keystone taxa, and prevented opportunistic pathogen expansion. Rather than restructuring the microbiota, this strain promoted resilience and functional integrity, consistent with the emerging paradigm of probiotics as ecological stabilizers (49). These results suggest that *B. infantis* YLGB-1496 is a safe and effective strategy for strengthening mucosal resilience and reducing the risk of common illnesses in early childhood, with broader implications for sustainable infection prevention and antimicrobial stewardship.

## 5 Conclusion

Based on the findings of this 12-week, double-blind, randomized, placebo-controlled trial, daily supplementation with *Bifidobacterium infantis* YLGB-1496 confers significant health benefits to healthy children aged 1–3 years by modulating gut microbiota ecology and host immune responses, without inducing major structural upheaval in the microbial community. The probiotic group exhibited a marked reduction in the incidence and severity of both respiratory and gastrointestinal illnesses, evidenced by fewer clinical visits, lower antibiotic use, and a decreased frequency of associated symptoms such as fever, cough, and diarrhea. These clinical improvements were underpinned by a targeted immunomodulatory effect, characterized by a reduction in pro-inflammatory markers like fecal IFN-γ, IL-1β, and calprotectin, a trend toward increased fecal IgA, and a significant decrease in salivary cortisol, suggesting a potential gut-brain axis benefit. Crucially, microbiota analysis revealed that the placebo group displayed an unfavorable trajectory toward dysbiosis, which is marked by a decline in beneficial short-chain fatty acid producers like, while the probiotic group maintained ecological stability. This preservation of keystone taxa associated with gut health, barrier integrity, and anti-inflammatory metabolite production highlights that the primary mechanism of *B. infantis YLGB-1496* is not wholesale restructuring but rather ecological resilience, preventing the drift toward a dysbiotic state. *B. infantis* YLGB-1496 proves to be a safe and effective intervention that supports pediatric health by stabilizing the gut ecosystem, fine-tuning immune function, and significantly reducing the burden of common childhood infections.

## Data Availability

The data presented in this study are publicly available. This data can be found here: https://www.ncbi.nlm.nih.gov/sra, accession PRJNA1371681.
